# Dental Squibs

**Published:** 1869-05

**Authors:** J. P. Holmes

**Affiliations:** Hazlehurst, Miss.


### DENTAL SQUIBS.

BY J. P. HOLMES, HAZLEHURST, MISS.

A very lively sympathy exists between the teeth and some other parts of the body, more intimate and extensive than would at first seem possible. Proof of this fact is frequently met with in our profession by careful attention to a proper diagnosis of all patients calling for operations. A patient coming into the office suffering with a severe pain in right superior first molar; upon examination, the tooth is found to be free from caries, tartar, or any thing that would indicate pain, yet the pain is there. Upon examining the teeth on opposite side, the first bicuspid, or molar, is found badly decayed, nerve exposed. Upon the extraction of this tooth, or destruction of its nerve, the pain ceases in the sound tooth. Here we have a sympathy existing between a decayed tooth and a sound one.

Facial neuralgia is very frequently produced and kept up by diseased teeth, vanishing upon the removal of the teeth. A diseased tooth will frequently cause severe pain in the face, neck, ear, eye, or throat, because all these parts are supplied by nerves derived from the same sources, or are more or less intimately connected. I have known a severe case of pleurodynia kept up by a diseased tooth until its removal, other remedies failing. Enlargement of the lymphatic ganglions of the neck, ulcers cf the chin, epilepsy, hysteria, dyspepsia, loss of sight, nervousness, sick headache, and various other affections, sometimes of a very obstinate and distressing character, are caused by diseased teeth. Dysmenorrhœa is cured sometimes by the removal of diseased teeth. Have had two practical cases of this kind recently.

Chills cured by the extraction of teeth.—A young lady called to have two teeth extracted—superior central incisors; had been broken off by a hook from a cow some four or five years since; administered gas and removed them without much force or trouble; patient left for her home a mile from town; on arriving there active hemorrhage set in, lasting five hours; she become frightened and sent for me; on arriving at her house found her in bed, very pale and much frightened; had bled nearly a quart of dark venous blood; washed sockets with tepid water and packed them with cotton saturated with Monsel's per-sulphate of iron, which checked the hemorrhage in a few moments; patient had been having chills for months and could not break them; has had no chill since; health much improved.

Sight restored by extraction of two teeth.—Mr. L., thirty years of age, came into my office three months since to have his teeth examined; said his right eye had been diseased for ten years; could not see out of it; pained him a great deal; on examination found first and second bicuspids on right side of superior maxillary (side that the eye was effected) decayed to the gums and both abscessed; informed him that these teeth ought to be removed, as I thought they were doing the mischief; he consented; extracted them and applied aconite and chloroform to the sockets, as they pained him very much; saw him a week since entirely well and sight restored.

Supernumerary teeth.—Mr. Wm. Fugate, a young man twenty-five years of age, called a few days since to have two teeth removed; on examination found a magnificent set of thirty-four permanent teeth, thirty-two in arch, regular, sound and in fine condition. Just back of and between the central and lateral incisors, were two supernumerary permanent teeth, erupted four years since; these teeth were inserted in palate bone, the crowns somewhat round and resembling the cuspid teeth ; roots more curved.

Extraction of teeth ordered by a physician for Dysmenorrhœa--Effects of Gas.—A lady, thirty years of age, was sent to me by her family physician, with orders to have all of her decayed teeth extracted; on examination found her mouth full of diseased teeth ; gave her the gas three times, giving more than usual; could only extract two at each administration, as she would push back in the chair with great force and rally in a few seconds, the gas seeming to have little or no effect; after extracting six teeth, badly diseased, one tooth having three distinct sacks on the roots, I discharged her, with instructions to call again in a few days and I would extract the rest. The gas had less effect on her than any patient I ever had. I attributed it to great nervous excitement and over-charged vessels with blood from arrest of menses. Will write you again about this patient. I am very much in the dark on this subject, but receive a good deal of light and instruction from the interesting pages of the Dental Register and *Cosmos*, two companions that I am very much attached to, and from which I derive a great deal of useful knowledge, as well as pleasure.

